# Unveiling the Role of Biomarkers in Cardiovascular Risk Assessment and Prognosis

**DOI:** 10.7759/cureus.51874

**Published:** 2024-01-08

**Authors:** Sumit Bhatnagar, Mohit Jain

**Affiliations:** 1 Medicine/Interventional Cardiology, Ram Krishna Dharmarth Foundation University (RKDF) Medical College Hospital & Research Centre, Bhopal, IND; 2 Cardiology, Liaquat National Hospital and Medical College (LNMC), Bhopal, IND

**Keywords:** micrornas (mirnas), natriuretic peptides, troponins, cardiovascular risk, biomarkers

## Abstract

Cardiovascular diseases (CVDs) remain a leading cause of global morbidity and mortality, necessitating innovative approaches for accurate risk assessment and prognosis. This review explores the evolving role of biomarkers in advancing cardiovascular risk evaluation and prognostication. Utilizing cardiac biomarkers that represent diverse pathophysiological pathways has the potential to enhance risk stratification for CVD. We delve into the intricate molecular signatures indicative of cardiovascular health, focusing on established biomarkers such as troponins, natriuretic peptides, and lipid profiles while also examining emerging candidates like microRNAs and inflammatory markers. This review provides a holistic perspective on the current landscape of cardiovascular biomarkers, offering insights into their applications in risk assessment and prognosis. In evaluating the risk and prognosis of heart failure (HF), the measurement of natriuretic peptides (B-type natriuretic peptide [BNP] or N-terminal pro-B-type natriuretic peptide [NT-proBNP]) or markers of myocardial injury (cardiac troponin I [TnI] or T [TnT]) has demonstrated utility. By elucidating the synergistic interplay between traditional markers and cutting-edge technologies, this work aims to guide future research endeavors and clinical practices, ultimately contributing to more effective strategies for risk assessment and prognosis of cardiovascular disease.

## Introduction and background

Cardiovascular diseases (CVDs) stand as a prominent global cause of mortality, claiming approximately 17.9 million lives annually and significantly impacting both health and healthcare expenses [1]. A continuous increase in the prevalence of CVD was reported, and this trend is projected to continue [2]. Globally, a 77.12% increase in CVD cases was reported from 1990 (31.31 million) to 2019 (55.54 million) [3]. It is estimated that by 2030, 23.6 million individuals will have died of CVD [2,4]. Traditional risk prediction models like the Framingham Risk Score are commonly employed to calculate an individual's risk for primary prevention of CVD; however, the score has limitations, such as reduced accuracy in some ethnic groups and a lack of inclusion of certain risk factors [5,6]. Nevertheless, there is a lack of effective tools for assessing risk in the context of the secondary prevention of CVD [5]. The diagnosis, risk stratification, and management of patients experiencing heart failure (HF) and acute coronary syndrome (ACS) have significantly benefited from the essential role of circulating biomarkers in ensuring prompt, reliable, and effective outcomes [2,5]. Recent studies have pinpointed multiple biomarkers from various biological pathways that are linked to cardiovascular (CV) risk and have the potential to provide prognostic information. Furthermore, the simultaneous utilization of multiple biomarkers has shown efficacy in the assessment of cardiovascular (CV) risk [5]. This narrative review provides an overview of CVD biomarkers that could be used in CV risk assessment and prognosis.

## Review

Definition and classification of biomarkers

According to the World Health Organization, biomarkers are any substance, structure, or process that can be measured in the body or its products and influence or predict the incidence of an outcome or disease [7]. Categorized according to their designated purposes for screening, diagnosis, or prognosis, biomarkers can be classified as diagnostic, prognostic, predictive, and pharmacodynamic biomarkers [5,8]. Diagnostic biomarkers facilitate the early detection of disease. A prognostic biomarker provides information on the likely course of the disease condition, whereas a predictive biomarker identifies individuals who are most likely to respond to a given therapy, hence helping tailor therapy. Lastly, the effect of a drug on the disease state is measured using pharmacodynamic biomarkers [8]. According to Morrow and de Lemos criteria, a biomarker should allow accurate and repeated measurement, be easy to obtain, and have a reasonable cost. A biomarker should provide added value over existing measures, and it must assist in clinical decision-making and enhance clinical care [5,8].

Cardiac biomarkers

Cardiac markers are naturally occurring substances that are released into the bloodstream in response to heart injury or stress [9]. The assessment of cardiac biomarkers through tests can aid in assessing an individual's risk of cardiovascular disease (CVD) and in monitoring and treating those suspected of experiencing acute coronary syndrome (ACS), cardiac ischemia, and heart failure (HF) [2]. Cardiac biomarkers can be classified based on various pathophysiological processes, such as biomarkers of myocardial injury, biomarkers of inflammation, biomarkers of plaque instability/rupture, biomarkers of platelet activation, biomarkers of neurohormonal activation, biomarkers of myocardial dysfunction or stress, biomarkers of microRNAs, and lipid biomarkers [10] (Table 1).

**Table 1:** Different biomarkers are associated with multiple pathophysiological processes cTn: Cardiac troponin, hs-cTn: High-sensitivity cardiac troponin, H-FABP: Heart-type fatty acid binding protein, hs-CRP: High-sensitivity C-reactive protein, GDF-15: Growth-differentiation factor-15, UA: Uric acid, PAPP-A: Pregnancy-associated plasma protein-A, MPO: Myeloperoxidase, MMPs: Matrix metalloproteinases, Lp-PLA2: Lipoprotein-associated phospholipase A2, sPLA2: Secretory phospholipase A2, sCD40L: Soluble cluster of differentiation 40 ligand, MR-proADM: Mid-regional-pro-adrenomedullin, BNP: B-type natriuretic peptide, ST2: Suppression of tumorigenicity 2 protein, ET-1: Endothelin-1, Gal3: Galectin-3, NRG-1: Neuregulin-1, Apo B: Apolipoprotein B, Non-HDL-C: Non-high density lipoprotein cholesterol, IL-6: Interleukin-6, LDL-C: Low‑density lipoprotein cholesterol

Pathophysiological Classification	Biomarkers	Description of Biomarkers
Biomarkers of myocardial injury	Cardiac troponin (cTn)	Cardiac troponin, a complex of proteins, inhibits muscle contraction [10]. Heart-specific cardiac troponin T (cTnT) and troponin I (cTnI) are sensitive markers for myocardial damage [10,11]. In acute myocardial infarction (AMI), both intact and degraded cTnI and cTnT are released [10,12]. The detection of cardiac troponin (cTn) in peripheral blood quantifies cardiomyocyte damage [10,12]. A major limitation is low sensitivity during AMI onset. This is attributed to a delayed rise in circulating troponin levels, necessitating repeated sampling over 6-9 hours for a substantial portion of patients.
	High-sensitivity cardiac troponin (hs-cTn)	These are newer-generation troponin assays [10]. hs-cTn assays are markers that assess ongoing myocardial injury in stable patients and even seemingly healthy populations [10,13].
	Heart-type fatty acid binding protein (H-FABP)	H-FABP, a low-molecular-weight protein (132 amino acids), is integral to myocardial fatty acid metabolism [10,14]. Abundantly present in cardiomyocytes, H-FABP is rapidly released into the cytosol during acute myocardial infarction (AMI) [10]. H-FABP could be a useful indicator for the early identification of high-risk patients in the general population.
Biomarkers of inflammation	High-sensitivity C-reactive protein (hs-CRP)	It is a nonspecific inflammatory marker that itself regulates atherothrombosis [10,15]. It is produced predominantly in the liver as part of the systemic response to inflammation [10]. Hs-CRP can detect lower levels of CRP (< 5mg/L) and classify patients into low, intermediate, and high-risk groups [10]. The European Society of Cardiology (ESC) guidelines recommend (class IIb) the use of hsCRP in patients with unusual or moderate cardiovascular risk profiles as a part of risk assessment [10,16]. Even though hsCRP is directly linked to cardiovascular events, recent research has affirmed its role as a predictor rather than a causal factor of cardiovascular disease (CVD).
	Growth-differentiation factor-15 (GDF-15)	GDF-15 is a divergent member of the transforming growth factor-β cytokine superfamily and is expressed by activated macrophages [10,17]. It is associated with cellular oxidative stress, ischemia, and strain [10]. Clinical studies have reported that GDF-15 is a strong predictor of cardiovascular events, all of which cause death. Clinical studies demonstrate that GDF-15 shows promise as a means for categorizing risk levels [10]. However, GDF-15 lacks specificity for cardiovascular disease (CVD) and has been observed to be elevated in various malignancies, including prostate, colon, and glial cancers.
	Fibrinogen	Fibrinogen, a liver-synthesized acute-phase protein, can surpass 7 mg/mL during episodes of acute inflammation [10]. Elevated fibrinogen levels are associated with an increased risk of incident cardiovascular disease (CVD) [10,18].
	Uric acid (UA)	Uric acid (UA) is the final product resulting from the breakdown of purines in the human metabolic process [10]. Elevated serum UA (even below the clinical threshold for hyperuricemia) may contribute to the development of CVD through an increase in oxidative stress, endothelial dysfunction, and inflammation [10,19].
	Interleukin-6 (IL-6)	Interleukin-6 (IL-6) is a cytokine that plays a role in inflammation and has cardiovascular effects as it regulates hypertrophy and apoptosis of cardiomyocytes [2]. An increase in cardiac IL-6 expression was observed in advanced HF, which indicates its role in prognosis [2]. Increased levels of IL-6 are linked to left ventricular dysfunction before diagnosis of HF, indicating its role as a risk marker for the initiation and progression of HF [2].
Biomarkers of plaque instability/rupture	Pregnancy-associated plasma protein-A (PAPP-A)	PAPP-A activates insulin-derived growth factor-1 (IGF-1), which induces inflammation and lipid uptake that can contribute to atherogenesis and plaque instability [10, 20]. Circulating PAPP-A is a promising biomarker for risk stratification of ACS and might be useful to predict increased coronary thin-cap fibroatheroma burden and plaque instability.
	Myeloperoxidase (MPO)	MPO is one of the major contributors to the formation and rupture of plaque [10]. Research has indicated that MPO functions as a circulating marker of inflammation in CVD [10,21].
	Matrix metalloproteinases (MMPs)	MMPs are a family of endopeptidases that are secreted by different inflammatory and tumor cells as zymogens and are subsequently activated by proteinases [10]. MMP-2, MMP-8 and MMP-9 are the proteases that lead to atherosclerotic plaque rupture and clinical events by degenerating structural components of the plaque matrix [10]. An elevated MMP-2 is associated with an increased rate of subsequent ischemic cerebrovascular events [10,22].
Markers of platelet activation	Lipoprotein-associated phospholipase A2 (Lp-PLA2)	Lp-PLA2 is also known as platelet-activating factor acetylhydrolase, and it is mainly produced by monocytes and macrophages [10]. Studies have demonstrated that Lp-PLA2 activity acts as a predictor of coronary artery disease and stroke independently [10]. Higher levels of Lp-PLA2 have been linked to a higher cardiovascular risk, regardless of other contributing factors [10,23].
	Secretory phospholipase A2 (sPLA2)	Studies have shown that elevated levels of sPLA2-IIA and sPLA2 activity are associated with increased incidence and recurrent cardiovascular events [10].
	Soluble Cluster of differentiation 40 ligand (sCD40L)	CD40L belongs to the tumor necrosis factor superfamily and is seen in various cell types, including immune cells and nonimmune cells [10]. The surface expressed CD40L is cleaved to form sCD40L [10,24]. sCD40L binds to receptors on the platelet surface, which leads to activation and secretion of the soluble form in a complex circle of modulation [10,24].
Biomarkers of neurohormonal activation	Copeptin	Copeptin is a C-terminal part of the precursor pre-provasopressin (pre-proAVP), which is released in the same amount as arginine vasopressin (AVP) [10]. Copeptin serves as a well-established biomarker for heart diseases and also stands as a predictor of mortality instead of AVP [10]. A recent study has shown that copeptin could predict the development of CAD and CV mortality, both in diabetics and non-diabetic patients [10,25]. There is a notable rise in copeptin levels in patients experiencing an acute myocardial infarction (AMI); however, it is not a result of direct cardiac release into the coronary circulation during AMI. The question of whether the heart actively contributes to the release of copeptin into the bloodstream remains a subject of debate.
	Mid-regional-pro-adrenomedullin (MR-proADM)	Adrenomedullin (ADM) is a potent vasodilator synthesized in the adrenal medulla, vascular endothelial cells, and the heart in response to physical stretch and specific cytokines [10]. ADM levels in the heart will be increased as a result of pressure and volume overload [10,26].
Biomarkers of myocardial dysfunction or stress	Natriuretic peptides	BNP (B-type natriuretic peptide) and its amino-terminal cleavage equivalent (NT-proBNP) are released into the circulation from the myocardium as a result of end-diastolic wall stress, which, in turn, results from an increase in volume or pressure [10]. A recent study revealed that NT-proBNP is independently linked to the occurrence of heart failure (HF) and enhances the prediction of HF risk beyond traditional risk factors. This holds true even among individuals with obesity [10].
	Suppression of Tumorigenicity 2 protein (ST2)	ST2 belongs to the interleukin-1 receptor family and manifests in two distinct forms: a transmembrane receptor known as ST2L, and a soluble decoy receptor referred to as ST2. Studies have shown that individuals diagnosed with acute myocardial infarction, acute heart failure, and chronic heart failure have shown correlations between increased plasma sST2 concentrations and a raised likelihood of mortality and nonfatal adverse cardiac events [10].
	Endothelin-1 (ET-1)	ET1, a powerful vasoconstrictor and hormone promoting fibrosis, is released by vascular endothelial cells, with levels that correlate to sheer stress and pulmonary artery pressure [10]. The C-terminal portion of pro-Endothelin-1 (CTproET1) is the more stable form of ET1 [10]. Research indicates that CT-proET-1 is linked to cardiovascular death and heart failure (HF) regardless of clinical variables in stable patients with coronary artery disease and acute myocardial infarction and displays a prognostic value comparable to BNP or NT-proBNP [10].
	Galectin-3 (Gal3)	Gal3 is a glycoprotein-binding protein that is secreted by activated cardiac macrophages [10]. In 2010, the Food and Drug Administration (FDA) sanctioned Gal-3 as a novel biomarker for stratifying the risk of heart failure (HF) [10].
	Neuregulin-1 (NRG-1)	NRG-1 is a paracrine growth factor that is released from endothelial cells and binds to a family of erythroblastic oncogene B (ErbB) receptors on nearby cardiac myocytes to promote cell survival, growth, and maintenance [10]. Research has demonstrated a correlation between increased levels of NRG-1, and HF, and CAD [10].
Biomarkers of micro ribonucleic acid (miRNAs)	Micro ribonucleic acid (miRNAs)	In the initial stages following a myocardial infarction, cardiac miRNAs become detectable, potentially decreasing the time required for diagnosis [10]. Multiple pathways that are affected by miRNA regulation include lipid metabolism, glucose homeostasis, vascular integrity, and endothelial cell function, which have significant involvement in CVD [10]. Increased serum levels of miRNAs like miR-1, miR-133a, miR-208a/b, and miR-499 are involved in acute coronary syndromes [10].
Lipid Biomarkers	Apolipoprotein B (Apo B)	It is the primary apolipoprotein of chylomicrons, very low-density lipoprotein (VLDL), intermediate-density lipoprotein, and low-density lipoprotein (LDL) particles [27]. apo B-100 is a hepatic-derived lipoprotein that is used in the measurement of LDL particle concentration [27]. High levels of apo B indicate a higher risk of CVD even when LDL-C or non-HDL-C levels are low, especially in individuals with metabolic syndrome and type 2 diabetes [27].
	Apolipoprotein A-I (Apo A1)	Apo A-I is the major apolipoprotein in the high-density lipoprotein cholesterol (HDLc) particles [27]. The level of HDLc is inversely proportional to the risk of CVD [27]. apo A-I potentially offers a more precise reflection of the "atheroprotective" capability of lipid metabolism compared to high-density lipoprotein cholesterol (HDL-C) [27].
	Apo B/A-I ratio	The apolipoprotein B/A-I ratio is used as a measure of proatherogenic to anti-atherogenic cholesterol [27]. This ratio is found to be strongly associated with CVD risk [27].
	Non-high density lipoprotein cholesterol (Non-HDL-C)	Non-HDL-C provides an estimate of cholesterol in the atherogenic particles, including intermediate-density lipoprotein, VLDL, lipoprotein (a), and LDL [27]. Recent guidelines emphasize the importance of this parameter for assessing CVD risk [27].
	Low‑density lipoprotein cholesterol (LDL-C)	Elevated levels of LDL-C are an important risk factor for atherosclerotic CVD [2]. A rapid increase in LDL-C plasma content indicates a significant risk of CVD [2].

Biomarkers for risk assessment of heart failure

The conventional HF risk prediction tool primarily focuses on traditional CV risk factors. There is a growing interest in considering blood-based biomarkers to improve risk prediction [20]. Elevated levels of routinely measured biomarkers associated with cardiac injury, stress, systemic inflammation, and indications of left ventricular hypertrophy (LVH) are linked to an increased risk of HF among otherwise healthy adults [28].

Natriuretic Peptides for Risk Assessment

Clinical studies have shown that natriuretic peptides may serve as predictors for the development of heart failure with reduced ejection fraction (HFrEF) [7]. The Framingham Heart Study (FHS) identified B-type natriuretic peptide (BNP) and the urinary albumin-to-creatinine ratio as biomarkers associated with predicting the onset of new heart failure [7,29]. Studies have shown that NT-proBNP, midregional pro atrial natriuretic peptide (proANP), high-sensitivity (hs) cardiac troponin T (TnT), cystatin-C, and urinary albumin excretion were also predictive for new-onset HF [7,30-32]. Aggregate evidence from studies reported the best predictive values of BNP and NT-proBNP, stronger than ANP and NT-proANP [7,33]. A meta-analysis demonstrated that the assessment of NT-proBNP concentrations strongly correlates with the emergence of new heart failure (HF) and enhances the prediction of coronary heart disease and stroke [34]. This research proposes that evaluating NT-proBNP concentrations could function as a versatile biomarker in innovative strategies that integrate HF into primary prevention for cardiovascular disease (CVD) [34]. Furthermore, another study indicated that regular physical activity is associated with a reduced likelihood of increasing NT-proBNP and hs-troponin over time [7,35].

Troponins for Risk Assessment

Elevated troponin levels demonstrated a connection with recognized risk factors for heart failure (HF), such as diabetes mellitus, left ventricular hypertrophy, chronic kidney disease, and increased natriuretic peptide levels. However, there was no association observed with prior myocardial infarction or coronary calcium [7,36-38]. Studies have shown a stronger relationship between the future risk of HF and elevated levels of troponins [7,39]. In a recent study, it was discovered that cardiac troponin independently predicts the risk of myocardial infarction or cardiovascular death, irrespective of cardiovascular risk factors and disease severity [40].

Renal Dysfunction Markers for Risk Assessment

Consistently, markers of renal dysfunction, such as creatinine or cystatin C, have been identified as reliable predictors of the onset of new heart failure (HF). In a thorough community study, the inclusion of kidney disease markers alongside traditional risk factors improved the precision of forecasting the 10-year risk of HF [41].

Emerging Markers for Risk Assessment

An examination of Gal-3's predictive utility for new-onset heart failure (HF) in the Framingham Heart Study (FHS) revealed that, in a fully adjusted model incorporating both BNP and Gal-3, there was a modest yet independent predictive value for the occurrence of new-onset HF [7,42]. Among individuals in the FHS, increased levels of soluble suppressor of tumorigenicity (sST2), hs-TnI, growth differentiation factor 15 (GDF-15), and BNP were found to be independently associated with the development of heart failure (HF) over an average follow-up period of 11 years [7]. The Atherosclerosis Risk in Communities study (ARIS Study) has shown that higher levels of ceruloplasmin have been linked to new-onset HF [43]. Moreover, inflammatory markers have been evaluated for predicting new-onset HF; the ABC study (Health, Aging, and Body Composition) demonstrated associations between IL-6, tumor necrosis factor-α, and CRP with the onset of new HF. Notably, when all three markers were included in the model, IL-6 emerged as the most robust market [7]. A recent scientific statement by the American Heart Association (AHA) proposes that, for predicting the onset of new heart failure, the measurement of natriuretic peptides (BNP or NT-proBNP) or markers of myocardial injury (TnI or TnT) alone contributes prognostic information beyond standard risk factors. Moreover, novel biomarkers such as sST2, Gal-3, GDF-15, and markers of renal function, whether employed individually or as part of a multimarker strategy, may prove valuable in risk stratification [7].

Biomarkers for Prognosis Assessment of Chronic Heart Failure

Biomarkers have been recognized as a valuable supplement to conventional clinical practices for evaluating the outlook for chronic HF [7]. In the context of chronic HF, both BNP and NT-proBNP have been identified as beneficial for determining prognosis. Increased concentrations of natriuretic peptides have been associated with the severity of HF, as assessed by parameters such as New York Heart Association (NYHA) class, elevated filling pressures, or more adverse hemodynamics [7,44-46]. The presence of circulating cTn serves as an additional valuable prognostic biomarker in chronic HF [7]. In contrast to certain biomarkers like natriuretic peptides, the concentration of the sST2 biomarker remains unaffected by factors such as age, renal function, or body mass index. Elevated levels of sST2 correlate with the prognosis of HF. Moreover, in individuals experiencing chronic ambulatory HF, increased concentrations of Galectin-3 (Gal-3) are moderately linked to mortality, although this association is not as robust as observed with other biomarkers [7,47].

Biomarkers for Prognosis Assessment of Acute Heart Failure

Assessing the prognosis of acute heart failure (HF) is a complex task for clinicians, with challenges in accurately determining the severity, leading to hospitalization of patients at lower risk and discharging those at higher risk [7,48]. The use of circulating biomarkers has become crucial for prognostic evaluation in acute HF. Research indicates that the initial presentation B-type natriuretic peptide (BNP) levels during dyspnea in an acute care setting serve as potent predictors of both short- and long-term outcomes [7,49-51]. Similarly, studies affirm that baseline N-terminal pro-B-type natriuretic peptide (NT-proBNP) is a robust predictor of outcomes [52,53]. Notably, BNP and NT-proBNP values measured before discharge and in the post-discharge period exhibit a strong correlation with outcomes compared to the levels at admission [54,55]. The levels of troponin provide additional prognostic information beyond what can be obtained from other clinical markers and findings during physical examinations. Therefore, it is recommended to include troponin levels as part of the early risk assessment in the initial evaluation of AHF [7]. Elevated levels of cardiac troponin I (cTnI) and cardiac troponin T (cTnT) both correlate with an unfavorable prognosis and are linked to compromised hemodynamics, a progressive decline in LV systolic function, and reduced survival. Similarly, the prognostic significance of sST2 is reported to be supplementary to that of NT-proBNP [7]. Consequently, individuals with elevated levels of both biomarkers exhibit the highest one-year mortality, whereas those with low values for both biomarkers experience the lowest mortality [7]. A recent scientific statement from the AHA suggests that for patients experiencing acute decompensated HF, assessing BNP or NT-proBNP and cTn levels at the time of presentation is beneficial for determining the prognosis or severity of the condition. In addition, in patients with acutely decompensated HF, measurement of biomarkers of myocardial injury or fibrosis is reasonable for additive risk stratification [7,56].

Novel Risk Model Markers for Cardiovascular Risk Prediction

Multiple guidelines recommend the use of a multivariable risk score to estimate the absolute risk of CVD. Conventional clinical risk scores such as pooled cohort equations (PCEs), the SCORE2 model, and the QRISK3 model provide an imprecise estimate of CHD risk. Imaging of subclinical atherosclerosis with computed tomography (CT) to detect coronary artery calcium (CAC) has been shown to be a potent predictor of future clinical CVD [57]. CAC is a measure of subclinical coronary atherosclerosis that reflects the cumulative exposure to cardiovascular risk factors over a lifetime. Recent guidelines recommend that selective use of a CAC score could be helpful in decision-making for primary atherosclerotic cardiovascular disease (ASCVD) prevention therapy [58]. Research has indicated that incorporating the coronary artery calcium score into a model based on traditional risk factors resulted in a statistically significant and clinically significant enhancement in distinguishing and categorizing CVD risk [57].

## Conclusions

Several cardiovascular disease biomarkers are presently accessible and serve clinical purposes as diagnostic, prognostic, or predictive indicators. Utilizing cardiac biomarkers that represent diverse pathophysiological pathways has the potential to enhance risk stratification for cardiovascular disease (CVD). In evaluating the risk and prognosis of heart failure (HF), the measurement of natriuretic peptides (BNP or NT-proBNP) or markers of myocardial injury (TnI or TnT) has demonstrated utility.
